# Air Damping Analysis of a Micro-Coriolis Mass Flow Sensor

**DOI:** 10.3390/s22020673

**Published:** 2022-01-16

**Authors:** Yaxiang Zeng, Remco Sanders, Remco J. Wiegerink, Joost C. Lötters

**Affiliations:** 1 Integrated Devices and Systems Group, University of Twente, P.O. Box 217, 7500 AE Enschede, The Netherlands; r.g.p.sanders@utwente.nl (R.S.); r.j.wiegerink@utwente.nl (R.J.W.); j.c.lotters@utwente.nl (J.C.L.); 2 Bronkhorst High-Tech B.V., Nijverheidsstraat lA, 7261 AK Ruurlo, The Netherlands

**Keywords:** Coriolis mass flow sensor, resonant sensors, mechanical dissipation, air damping

## Abstract

A micro-Coriolis mass flow sensor is a resonating device that measures small mass flows of fluid. A large vibration amplitude is desired as the Coriolis forces due to mass flow and, accordingly, the signal-to-noise ratio, are directly proportional to the vibration amplitude. Therefore, it is important to maximize the quality factor Q so that a large vibration amplitude can be achieved without requiring high actuation voltages and high power consumption. This paper presents an investigation of the Q factor of different devices in different resonant modes. Q factors were measured both at atmospheric pressure and in vacuum. The measurement results are compared with theoretical predictions. In the atmospheric environment, the Q factor increases when the resonance frequency increases. When reducing the pressure from 1 bar to 0.1 bar, the Q factor almost doubles. At even lower pressures, the Q factor is inversely proportional to the pressure until intrinsic effects start to dominate, resulting in a maximum Q factor of approximately 7200.

## 1. Introduction

Coriolis mass flow sensors measure true mass flow independent of fluid properties [1]. A Coriolis flow sensor consists of a suspended channel that is brought into vibration. A fluid flow inside the vibrating channel will experience Coriolis forces that are proportional to the product of the mass flow $\Phi_{m}$ inside the channel and the local angular velocity Ω of the channel:

(1) $$\begin{array}{c} \frac{F}{\Delta L}=-2\Omega \times \Phi_{m} \end{array}$$

where F/ΔL represents the Coriolis force per unit length that is acting on the channel. Equation (1) shows that the Coriolis forces are proportional to the angular velocity Ω. Therefore, the suspended channel is usually actuated at its resonance frequency to achieve a high amplitude of the angular velocity. For power-efficient actuation, the damping of the micro-Coriolis channel due to, e.g., the surrounding air, needs to be minimized. Furthermore, for a given actuation amplitude, the vibration induced by the Coriolis forces can be enhanced by designing the sensor such that the resonance frequencies of the actuation mode and the Coriolis mode are close together. However, the extent to which this can be achieved is limited by the quality factor Q. Too much damping can cause the sensitivity of the sensor to become dependent on the quality factor, resulting in a sensitivity that is dependent on air pressure [2]. In [2], it was shown that a ratio between the two resonance frequencies of 1.4 requires a quality factor of at least 30.

Various mechanisms can be distinguished that cause damping on the resonating channel of a Coriolis flow sensor, such as air drag, squeeze-film damping [3], thermo-elastic damping [4], and acoustic anchor loss [5]. These mechanisms can be divided into two categories: internal damping, which is originating from the materials that form the device, and external damping, which is introduced by the fluid environment. For a micro-Coriolis flow sensor that works at atmospheric pressure, air drag is the major source of damping, as will be shown in this paper. Operation at atmospheric pressure is preferred because this eliminates the need for the special vacuum packaging of the device.

Many studies have been reported on air drag in resonating microstructures operating in viscous fluids. Kokubun et al. [6] introduced a model in which the suspended structure is regarded as a chain of spheres to analyze the damping of a quartz oscillator. Hosaka et al. [7,8] improved this model and validated the model on different structures with different resonant modes [9]. The sphere model can qualitatively describe the air drag in a resonating structure. However, it does not provide a method to determine the diameter of the sphere. Sader [10] proposed an analytical model to describe the frequency response of the drag force experienced by a cantilever beam with a circular or thin rectangular cross section. This model was validated by Bergaud et al. [11] and Maali et al. [12]. Based on these studies and measurements, the micro-cantilever beam was investigated as a tool for measuring the density and viscosity of fluids [13,14,15].

However, most of the reported work on air damping is about straight beams with rectangular cross section or thin plates with a thickness that is much smaller than the width and height [16,17]. The researched beams or thin plates are placed either in free space [18,19] or very close to the silicon body [20,21]. Little research has been conducted on suspended structures with a more complicated geometry and cross section. The influence of small attached structures, such as capacitive readout electrodes, is also out of the scope of existing papers.

In this paper, the effect of air damping on micro-Coriolis mass flow sensors is investigated. The paper is organized as follows. First, in Section 2, the structure of a micro-Coriolis flow sensor is briefly introduced. Next, in Section 3, the theory about air drag for a micro beam in air at atmospheric pressure and in vacuum is presented. The additional damping due to capacitive readout electrodes is derived by finite element simulations. Section 4 explains the measurement setup for measurement of the Q factor at atmospheric pressure and in vacuum. Section 5 presents the measurement results and makes a comparison with the theoretical prediction presented in Section 3. Finally, the conclusions are presented in Section 6.

## 2. Micro-Coriolis Flow Sensor

**Figure 1:**
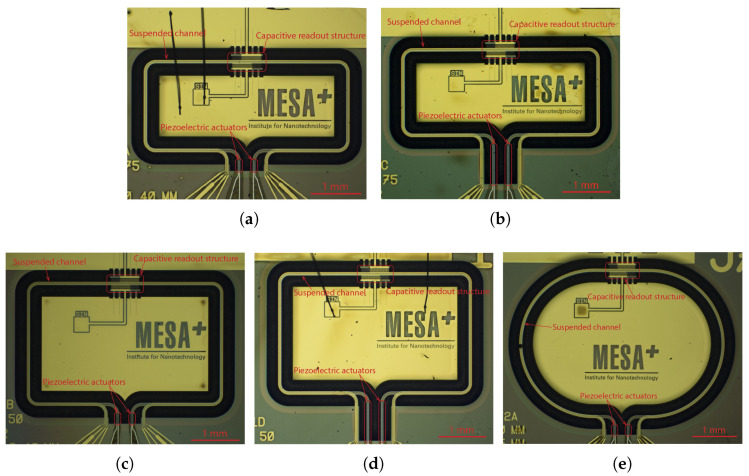
Optical microscope photographs (**a**–**e**) of the 5 micro-Coriolis mass flow sensor devices that were used for Q factor measurements. The position of the capacitive readout structures and piezoelectric actuators are indicated. Using these 5 different devices allowed the measurement of quality factor at 20 different resonance frequencies in the range from 1.8 kHz to 16 kHz.

**Figure 2:**
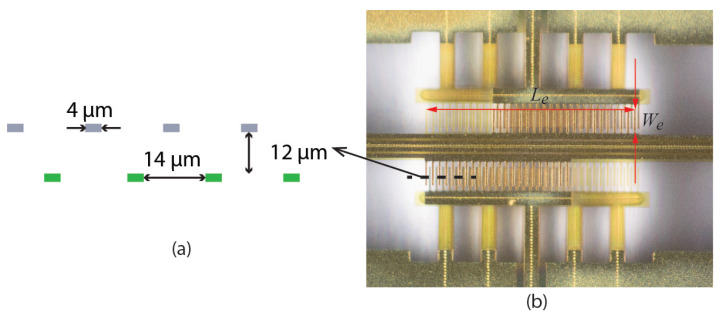
Schematic cross section (**a**) and close up photograph (**b**) of a capacitive readout structure. Two capacitors, one at each side of the channel, are formed by interdigitated combs. The combs shown in gray in the cross section are attached to the vibrating channel while the combs shown in green are attached to the silicon substrate. $L_{e}=580\mu m$, $W_{e}=205\mu m$.

Figure 1 shows photographs of the micro-Coriolis flow sensor chips that were used for this paper. A rectangular loop of a microfluidic channel is suspended from the bulk silicon at one side. Capacitive readout structures are placed at the other side of the rectangle. Figure 2 shows a close up photograph of a capacitive readout structure. The rectangular channel loop is suspended approximately 200 μm above the silicon substrate, while the channel is 70 μm wide and 35 μm high. Because the channel width is significantly smaller than the distance to the substrate, squeezed film effects can be neglected. The fabrication method of the sensor was described in [22].

**Figure 3:**
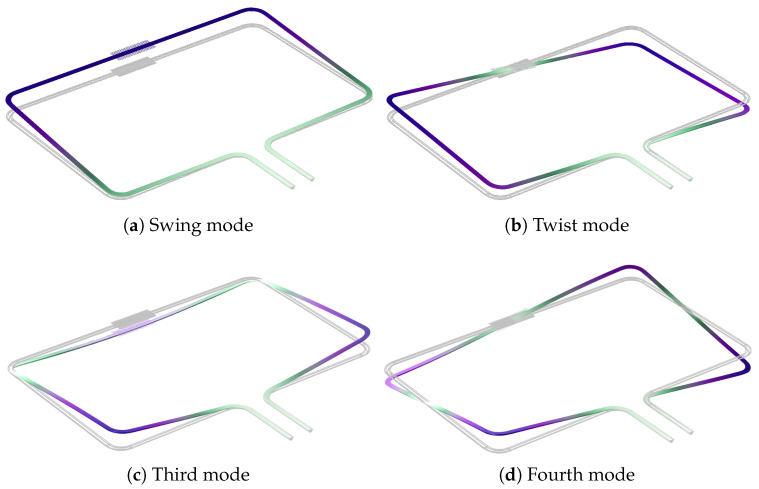
Mode shapes (**a**–**d**) for the four actuation modes that are used in this paper. The swing mode (**a**) and twist mode (**b**) are usually used for mass flow sensing.

The suspended channel can be actuated into different resonant modes, as shown in Figure 3. In this paper, the channels are actuated by piezoelectric actuators as presented in [22]. The modes shown in Figure 3a,b are used for Coriolis mass flow sensing. In the case of swing mode actuation, the Coriolis mode is the twist mode, and vice versa. The Coriolis mode is actuated by the Coriolis forces generated by the fluid flowing inside the channel. The mass flow is measured by measuring the ratio between the amplitudes of the actuation mode and Coriolis mode [2].

**Figure 4:**
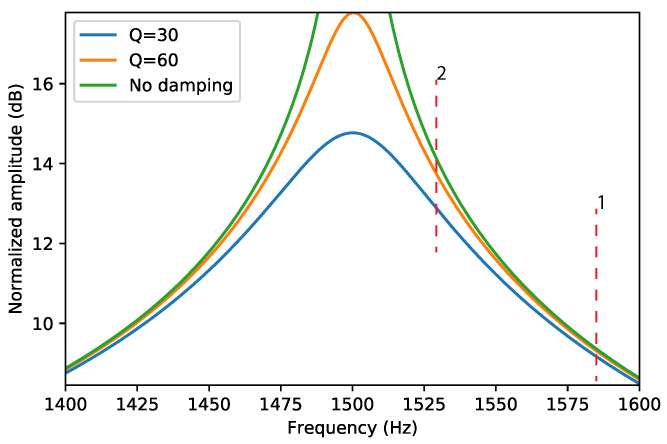
Typical frequency response of a harmonic oscillator which represents the Coriolis mode close to the resonance frequency and at different values of the quality factors. In this example, the resonance frequency is set to 1500 Hz. The two red dashed lines indicate possible values of the resonance frequency of the actuation mode. In situation 2, the sensitivity is higher, however at the expense of an increased dependence on the value of the quality factor.

The Coriolis mode is actuated at the resonance frequency of the actuation mode. It was shown [2] that the sensitivity of the sensor increases if the resonance frequencies of the actuation mode and Coriolis mode are closer together. However, if the quality factor is not sufficiently high this may result in a sensitivity that is dependent on the quality factor. This is illustrated in Figure 4, which shows the frequency response of the Coriolis mode for different Q factors. The red dashed lines indicate two possible resonance frequencies of the actuation mode. At actuation frequency 1, the three frequency responses are very close together, and a change in Q factor from 60 to *∞* has little effect on the amplitude of the Coriolis motion. For actuation frequency 2, we see that the sensitivity is higher; the amplitude of the Coriolis motion will be larger. However, a significant difference can be seen between the responses for different quality factors, which will make the sensitivity dependent on the amount of damping. Thus, the resonance frequency difference between the actuation and Coriolis modes need to be large enough so that a change in the Q factor of the Coriolis mode will not influence the flow measurement result. Understanding the factors that influence the Q factor helps to design Coriolis flow sensor devices that give a stable Coriolis mode amplitude while having optimal sensitivity.

**Figure 5:**
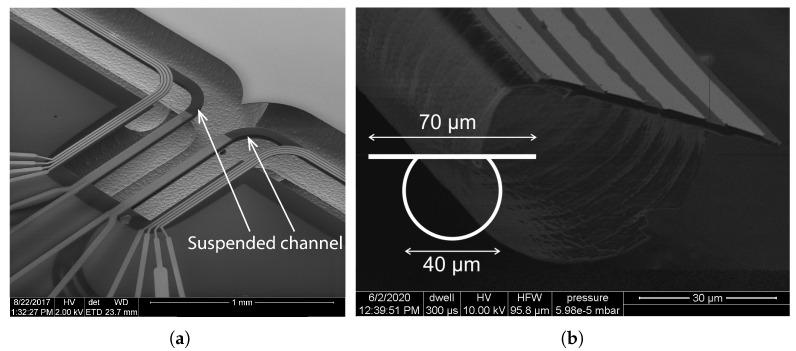
(**a**) SEM image of the suspended channel in a micro-Coriolis mass flow sensor, and (**b**) a cross sectional image of the channel with a schematic drawing showing the dimensions of the cross section.

Figure 5a shows a SEM photograph of the suspended channel of the device in Figure 1b. The channel is suspended roughly 200μm above the silicon substrate. Figure 5b shows a cross sectional SEM image of channel. The cross section of the channel can be approximated by a 270 ${}^{\circ }$ arc of 40 μm diameter combined with a flat beam with a width of 70 μm as indicated by the schematic drawing in the figure.

## 3. Theory

Damping mechanisms can be classified into two categories: extrinsic and intrinsic. Intrinsic damping stems from the structure and material that form the device, such as thermal elastic damping and support loss. Extrinsic damping is induced by the environmental fluid and can be minimized by operating the device in vacuum. Although a micro-Coriolis mass flow sensor consists of a suspended channel with fluid flow inside, the fluid inside the channel causes very little damping [23]. The relation between the overall quality factor $Q_{tot}$, the contribution of the intrinsic damping $Q_{intrinsic}$ and the contribution of the extrinsic damping $Q_{external}$ can be expressed as:

(2) $$\frac{1}{Q_{tot}}=\frac{1}{Q_{intrinsic}}+\frac{1}{Q_{external}}$$

When the extrinsic damping is much larger than the intrinsic damping, $Q_{tot}$ approximately equals $Q_{external}$.

Different models are used to describe the extrinsic damping of vibrating structures at different pressure ranges [24]. At low pressure, if the mean free path of the gas molecules is comparable or larger than the characteristic size of the vibrating structure, the environmental fluid cannot be regarded as continuous medium. At higher pressure, the environmental medium can be seen as a continuu,m and it can be described by the Navier–Stokes equations. The Knudsen number is used to determine the model that should be used to describe the fluid environment. It represents the ratio between the molecular mean free path λ and the characteristic size $L_{C}$ of the vibrating structure [24]:

(3) $$K_{n}=\frac{\lambda }{L_{c}}=\frac{k_{B}T}{\sqrt{2}\pi d^{2}pL_{c}}$$

where $k_{B}$ is the Boltzmann constant, *T* is the thermodynamic temperature, *d* is the hard-shell diameter of a gas molecule, and *p* is the pressure. The width of the suspended channel is 70 μm, which is used as the characteristic length $L_{c}$. Table 1 shows the characteristics of different flow regimes and the corresponding pressure ranges.

### 3.1. Continuum Regime

In the continuum regime, the Reynolds number Re of the fluid flow around the channel is expressed as:

(4) $$Re=\frac{\rho u_{c}L_{c}}{\mu }=0.058$$

where ρ is the density of air at atmospheric pressure, $u_{c}$ is the characteristic velocity of the channel, and μ is the dynamic viscosity of air. With a resonance frequency of 2 kHz and a vibration amplitude of 1 μ m, $u_{c}=1.26\times 10^{-2}\;m\;s^{-1}$. Equation (4) shows that Re is very small, thus the air flow around the channel is laminar.

The fundamental equations of fluid mechanics show that in the laminar flow region, the hydraulic force acting on a body oscillating with a velocity u in a fluid can be generally expressed as [25]:

(5) $$\begin{array}{c} \begin{array}{cc} F_{l}= & F_{ip}+F_{op}\\ = & \beta_{1}u+\beta_{2}\frac{du}{dt} \end{array} \end{array}$$

where $F_{l}$ is the hydraulic force per unit length, which can be divided into a component $F_{ip}$ that is in phase with the velocity and a component $F_{op}$ that has a π/2 phase shift with the velocity. The latter appears as an additional mass of the vibrating structure. For an oscillating body in air, this additional mass is small, and it does not significantly influence $Q_{tot}$. The coefficients $\beta_{1}$ and $\beta_{2}$ are independent of velocity u.

An oscillating body inside a viscous fluid will excite a transverse wave. The amplitude of this wave drops off exponentially with the distance from the oscillating body and can be described by the penetration depth δ [25]:

(6) $$\delta =\sqrt{\frac{2\mu }{\rho \omega }}$$

where ω represent the angular frequency of oscillation.

An important dimensionless number β for an oscillating body is related to the ratio between δ and the characteristic size of the body:

(7) $$\beta =\frac{L_{c}^{2}}{2\delta^{2}}=\frac{\rho \omega L_{c}^{2}}{4\mu }$$

The expression for β is similar to the expression for the Reynolds number and is sometimes also referred to as the Reynolds number [26], except that the linear velocity $u_{c}$ is replaced by the product of the angular velocity and the characteristic length $\omega L_{c}$. With a vibration frequency of 2 kHz, the penetration depth δ=49.1μm so that β=1.02.

Kokubun et al. proposed a model in which the resonating body is represented by a string of spheres [6]. Spheres are used because the problem of flow around a sphere is well solved. This model was further developed and experimentally validated [8,9]. Hosaka et al. [8] show that for a beam with width $L_{c}$, the coefficient $\beta_{1}$ in Equation (5) can be expressed as:

#### 3.1.1. Sader’s Model

Sader gave a detailed theoretical analysis of the frequency response of a cantilever beam in a viscous fluid [10]. This model was then validated on micro-cantilevers in liquid and gas environments [11,12,27]. Sader’s model was based on a theoretical analysis of a round cylinder [28].

For a micro-Coriolis flow sensor working in air only the real part of $F_{l}$ in Equation (5) is important because only this real part influences the Q factor and the extra mass induced by the imaginary part is small in comparison to the mass of the channel. Maali et al. [12] gave an expression for $\beta_{1}$:

(8) $$\beta_{1}=\frac{\pi }{4}\rho L_{c}^{2}\omega \Gamma^{\prime }$$

where $\Gamma^{\prime }$ is the imaginary component of the hydrodynamic function of the moving beam. For a value of β as given by Equation (7) in the range 1<β<1000, a relatively simple expression can be found for $\Gamma^{\prime }$ [12]:

(9) $$\begin{array}{c} \begin{array}{cc} \Gamma^{\prime }= & b_{1}\sqrt{\frac{1}{2\beta }}+b_{2}\frac{1}{2\beta } \end{array} \end{array}$$

with $b_{1}=3.8$ and $b_{2}=2.7$.

Inserting (9) into (8) gives the following expression for $\beta_{1}$:

(10) $$\beta_{1}=\frac{b_{1}}{4}\pi L_{c}\sqrt{2\rho \mu \omega }+\frac{b_{2}}{2}\pi \mu$$

#### 3.1.2. Damping Due to the Capacitive Readout Fingers

Each capacitor in the capacitive readout consist of two sets of fingers as shown in Figure 2. One set of fingers is attached to the silicon chip, and the other set of fingers moves with the suspended channel. Due to stress resulting from the fabrication process, the moving fingers are around 12 μm higher than the fixed fingers. The fingers are 4 μm wide and have a distance of 14 μm between each other. The distance between fingers is smaller than δ given by Equation (6), which indicates that the air between the fingers moves together with the fingers. Thus, Equation (10) cannot be used to calculate the drag force acting on individual fingers.

**Figure 6:**
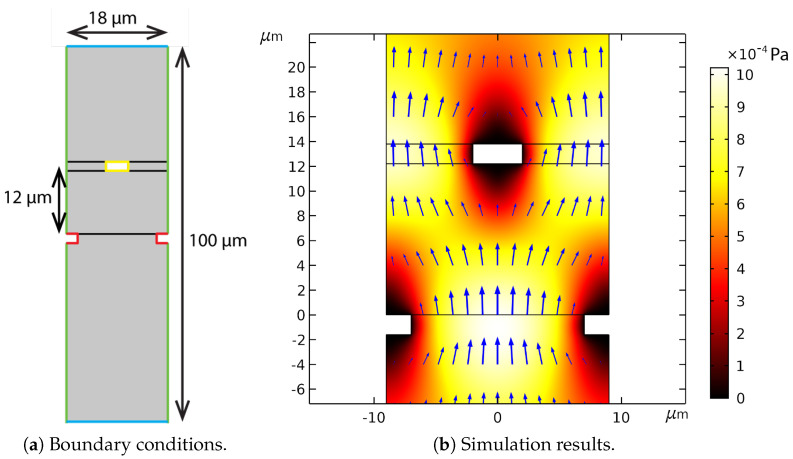
The domain of the 2D simulation (**a**) and the simulation result (**b**) of flow around the capacitive readout fingers. The gray area in (**a**) is the domain where fluid flow was simulated. The green lines indicate a symmetrical boundary condition. The blue lines indicate an open boundary condition. The red and yellow lines represent no-slip walls. The yellow rectangle represents a moving finger. The red lines represent the fixed fingers. The black lines separate the different domains of the moving mesh. The simulation results (**b**) show the flow and pressure when the moving finger has moved to the highest point and the velocity is zero. The color legend in (**b**) indicates the pressure. The blue arrows represent the flow velocity.

A finite element simulation with COMSOL Multiphysics was used to determine the drag induced by the fingers. A 2D model was used to simulate flow inside the cross sectional plane of a single moving finger as shown in Figure 6a. Both laminar flow and creeping flow were used as physical model. The velocity is far below speed of sound, so the flow was set to be incompressible. The top and bottom boundaries shown in red were set as an open boundary. The pressure at these boundaries was set to atmospheric pressure. The symmetric boundary condition was used for the green boundaries, where the flow velocity perpendicular to the boundary was set to zero. The yellow and red lines indicate the no slip wall boundary condition. The moving finger marked in yellow vibrates in the vertical direction. The moving finger is modeled as a motion of the fluid domain boundary. The deforming domain was simulated with the moving mesh method. The black lines in the figure are the boundaries of the moving meshes. Figure 6b shows the flow and pressure when the moving finger has moved to its highest point. The velocity of the finger is zero while there is still a flow. This indicate that there is a delay between the velocity and the fluid force.

**Figure 7:**
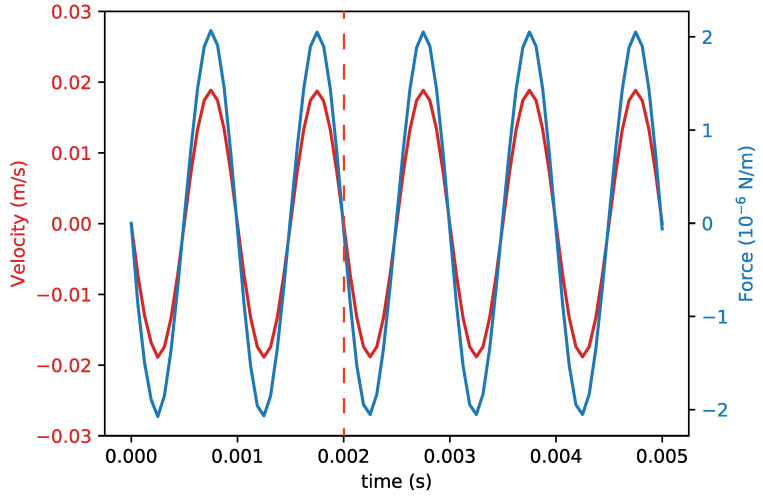
Velocity and simulated drag force as a function of time in the first 0.005 s. The data at the right hand side of the red dashed line was used for calculating $\beta_{1}$.

Figure 7 shows the velocity of the finger and the resulting drag force as a function of time from the creeping flow physical model, which is very similar to the result from the laminar flow physical model. This is as expected because the Reynolds number is very small. The vibration frequency and amplitude were set to 2 kHz and 0.5 μm, respectively. There is only a small delay between the velocity and the force, indicating that the $F_{op}$ component in Equation (5) is relatively small. The coefficients $\beta_{1}$ were extracted by fitting the force curve with a sinusoidal function. The time dependent simulation was started with the initial condition that both the fluid and the finger are at rest. Therefore, the first two oscillation periods were not used in the calculation of the coefficients.

**Figure 8:**
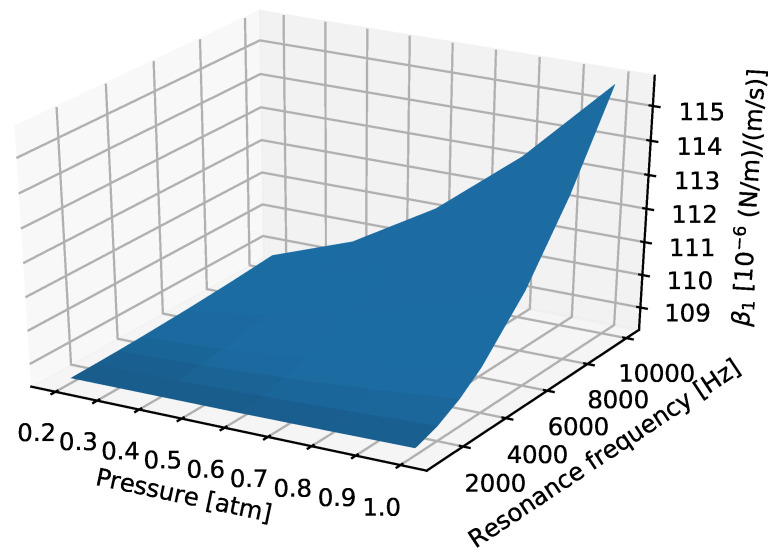
$\beta_{1}$ as the function of resonance frequency and environmental pressure.

Further simulations were performed with the creeping flow model to save computing time. Figure 8 shows the coefficient $\beta_{1}$ for a single finger, denoted as $\beta_{f}$, as a function of vibration frequency and pressure. The pressure of the environment influences $\beta_{f}$ because of the change in the density of the air, but we see that the value of $\beta_{f}$ only varies slightly over the presented pressure and vibration frequency ranges. Therefore, in our calculations, we will use a value of $1.1\times 10^{-4}$ (N/m)/(m/s) for each finger. This result is not surprising as the damping due to the capacitive readout fingers is similar to the squeezed film damping of a perforated plate, which is also independent of environmental pressure and vibration frequency [3].

The simulation gives the value of $\beta_{f}$ for a single infinitely long finger. However, in the actual devices the finger has a finite length, and it is connected to the suspended channel. In this paper, the value of $\beta_{1}$ for the channel segment with fingers is estimated by taking the sum of ${\left(\beta_{1}\right)}_{bare\;channel}$ from Equation (10) and the contribution per unit channel length of the fingers:

(11) $${\left(\beta_{1}\right)}_{capacitive\;readout}={\left(\beta_{1}\right)}_{bare\;channel}+\beta_{f}W_{e}/d_{e}$$

where $W_{e}$ is the length of the fingers as shown in Figure 2, so that $\beta_{f}W_{e}$ is the drag force divided by velocity for one finger, and $d_{e}$ is the center to center distance between the fingers.

### 3.2. Molecular Regime

In the molecular regime, the damping is determined by the pressure difference between the front and back side of the moving body. This difference is due to the collisions between gas molecules and the moving surfaces. The velocity distribution of the gas molecules is described by a Maxwell–Boltzman distribution. Christian [29] derived the pressure difference between the two sides of a moving flat plate:

(12) $$P_{d}=4\sqrt{\frac{2}{\pi }}uP\sqrt{\frac{M_{m}}{R_{0}T}}$$

with *u* being the velocity of the plate, *P* the pressure, $R_{0}$ the universal gas constant, *T* the absolute temperature, and $M_{m}$ the molecular mass, which is around 28.79 g ${\mathrm{mol}}^{-1}$ for air.

Thus, $\beta_{1}$ for a flat resonant beam can be expressed as:

(13) $${\left(\beta_{1}\right)}_{rectangular}=4\sqrt{\frac{2}{\pi }}L_{c}P\sqrt{\frac{M_{m}}{R_{0}T}}$$

and for a moving body with circular cross section, we have [29]:

(14) $${\left(\beta_{1}\right)}_{circular}=\frac{\pi }{4}{\left(\beta_{1}\right)}_{rectangular}$$

### 3.3. Quality Factor Calculation

The amount of air drag can be determined by measuring the quality factor of a resonating Coriolis mass flow sensor. The quality factor *Q* of a resonator is defined as:

(15) $$Q=2\pi \frac{E_{s}}{E_{d}}$$

where $E_{s}$ and $E_{d}$ represent the energy stored and energy dissipated per cycle, respectively.

The velocity of a channel segment at position *x* along the channel, when excited at a resonance frequency, can be expressed as:

(16) u(t)=D(x)sin(ωt)

where D(x) is the maximum velocity of the channel segment.

Thus, the stored mechanical energy can be derived by the integration of the maximum kinetic energy along the length of the channel:

(17) $$E_{s}=\int^{L}\frac{1}{2}\rho_{l}D{\left(x\right)}^{2}dx$$

where $\rho_{l}$ is the mass per unit length of the channel, which was calculated from the cross section of the channel. Using (5), the energy dissipated every cycle can be expressed as:

(18) $$\begin{array}{c} \begin{array}{cc} E_{d}= & \int^{L}\int_{0}^{T}F_{l}u\left(t\right)dtdx\\ = & \int^{L}\int_{0}^{T}\beta_{1}u{\left(t\right)}^{2}dtdx\\ = & \int^{L}\int_{0}^{T}\beta_{1}\sin{\left(\omega t\right)}^{2}D{\left(x\right)}^{2}dtdx\\ = & \frac{1}{2}T\int^{L}\beta_{1}D{\left(x\right)}^{2}dx \end{array} \end{array}$$

where T=2π/ω is the period of one cycle. By integrating $E_{s}$ and $E_{d}$ over the length of the channel, the total mechanical energy stored and total energy dissipated can be calculated.

The damping induced by the capacitive readout combs is different in different modes. Figure 3 shows the first four modes of a micro-Coriolis flow sensor. The motion of the readout combs is much higher in the swing and third mode than in the twist and fourth mode, so the associated damping will also be higher.

The stored mechanical energy and dissipated energy of the capacitive readout structure and the channel can be expressed separately in Equations (17) and (18):

(19) $$\begin{array}{c} \begin{array}{cc} E_{s}= & \frac{1}{2}{\left(\rho_{l}\int^{L}D{\left(x\right)}^{2}dx\right)}_{bare\;channel}+\\ & \frac{1}{2}{\left(\rho_{l}\int^{L}D{\left(x\right)}^{2}dx\right)}_{capacitive\;readout} \end{array} \end{array}$$

(20) $$\begin{array}{c} \begin{array}{cc} E_{d}= & {\left(\frac{\pi }{\omega }\beta_{1}\int^{L}D{\left(x\right)}^{2}dx\right)}_{bare\;channel}+\\ & {\left(\frac{\pi }{\omega }\beta_{1}\int^{L}D{\left(x\right)}^{2}dx\right)}_{capacitive\;readout} \end{array} \end{array}$$

The results are then inserted in Equation (15) to calculate the Q factor. The calculation can be simplified by defining the following ratio:

(21) $$R_{kinetic}=\frac{{\left(\int^{L}D{\left(x\right)}^{2}dx\right)}_{capacitive\;readout}}{{\left(\int^{L}D{\left(x\right)}^{2}dx\right)}_{bare\;channel}}$$

In this paper, D(x) is obtained from finite element simulations using COMSOL Multiphysics^®^. With the ratio $R_{kinetic}$, the expression for the *Q* factor is written as:

(22) $$Q=\omega \frac{{\left(\rho_{l}\right)}_{bare\;channel}+{\left(\rho_{l}\right)}_{capacitive\;readout}R_{kinetic}}{{\left(\beta_{1}\right)}_{bare\;channel}+{\left(\beta_{1}\right)}_{capacitive\;readout}R_{kinetic}}$$

with ${\left(\beta_{1}\right)}_{bare\;channel}$ from Equation (10) and ${\left(\beta_{1}\right)}_{capacitive\;readout}$ from Equation (11).

### 3.4. Quality Factor Estimation

An estimate for Q can be obtained without knowing $R_{kinetic}$ because $R_{kinetic}$ is smaller than one for all devices in all vibration modes. By ignoring the term with $R_{kinetic}$, Q can be expressed as:

(23) $$\begin{array}{c} \begin{array}{cc} Q= & \frac{\omega \rho_{l}}{\beta_{1}} \end{array} \end{array}$$

In the continuum regime, by inserting Equations (8) and (9) into Equation (23), we obtain:

(24) $$\begin{array}{c} \begin{array}{c} Q=\frac{\omega \rho_{l}}{\frac{b_{1}}{4}\pi L_{c}\sqrt{2\rho \mu \omega }+\frac{b_{2}}{2}\pi \mu } \end{array} \end{array}$$

This estimation is more precise for the twist and fourth mode because, in these two modes, the value of $R_{kinetic}$ is very small.

The damping induced by the capacitive readout combs is ignored when calculating the Q factor in high vacuum. By inserting Equation (13) into Equation (23), the Q factor in the molecular flow regime is written as:

(25) $$Q=\frac{\rho_{l}\omega }{L_{c}P}\sqrt{\frac{R_{0}T}{2\pi M_{m}}}$$

A summary of the parameters involved in simulations and calculations is shown in Table 2.

## 4. Measurement Method

To validate the expressions (24) and (25) found for the quality factor, two sets of measurements were performed. Firstly, measurements were performed at atmospheric pressure to evaluate the influence of mode shape and resonance frequency. Secondly, measurements were performed in a vacuum chamber to investigate the influence of pressure.

### 4.1. Mode Shape and Resonance Frequency Measurement

**Figure 9:**
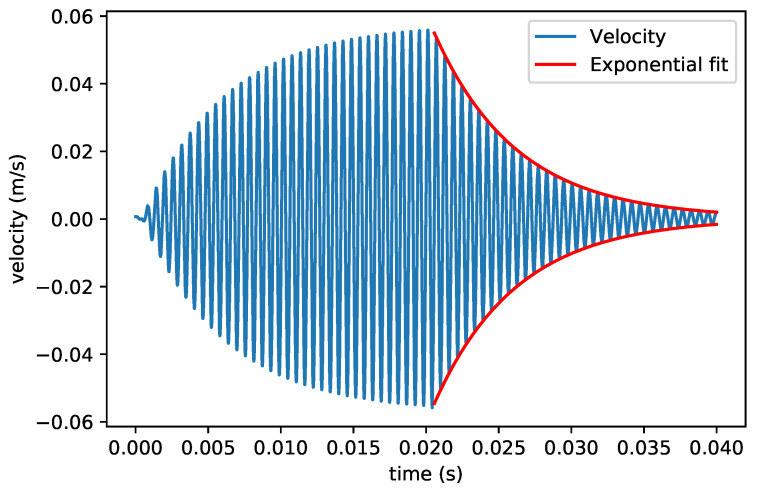
Measured velocity of the vibrating channel and exponential fit of the ring down process. This particular measurement was performed with the device from Figure 1d in the swing mode. The actuation voltage was applied at 0s and stopped after 0.022 s.

In this measurement, the 5 different micro-Coriolis mass flow sensor devices were actuated in the 4 modes that are indicated in Figure 3. The Q factors at atmospheric pressure are between 30 and 200. Therefore, ring down measurements were used to determine the Q factor. A Polytec MSA-400 laser Doppler vibrometer was used to record the instantaneous velocity of the vibrating sensor channel as a function of time [30]. Sinusoidal actuation voltages at the resonance frequency of the measured mode were applied to the integrated piezoelectric actuators until a sufficiently large vibration amplitude had developed. Figure 9 shows a typical measurement result, where the velocity amplitude of the channel first increases due to the applied actuation voltages and then decays once the actuation has stopped. The Q factor is obtained from an exponential fit of the decay in amplitude.

The actuation method may also induce extra damping mechanisms. For example, if the channels are actuated by Lorentz force [2], Eddy currents may cause extra energy dissipation. In this paper, piezoelectric actuators were used in order to minimize such effects. When measuring the Q factor of the swing mode and the third mode, the two signals applied to the two piezoelectric actuators (see Figure 1) are identical. When measuring the Q factors of the twist and fourth mode, the two signals applied to the two actuators have the same amplitude but a 180 ${}^{\circ }$ phase difference.

### 4.2. Rarefied Environment Measurement

**Figure 10:**
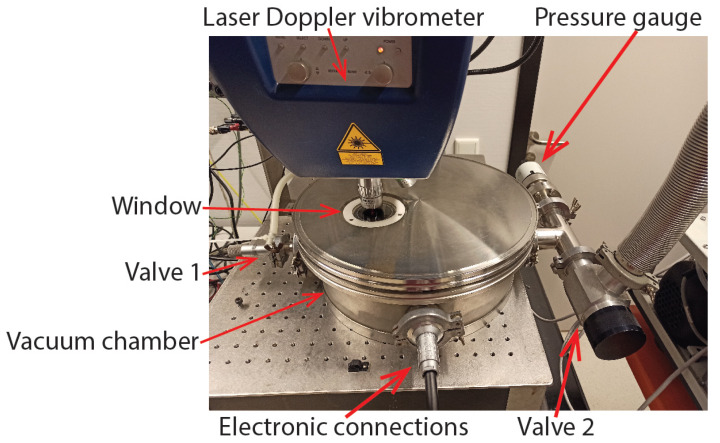
Photograph of the vacuum measurement setup. The laser Doppler vibrometer is measuring through the window.

To measure the Q factor as a function of pressure, separate measurements were performed for the continuum regime and free molecular regime. Figure 10 shows a photograph of the vacuum chamber with the MSA-400 laser Doppler vibrometer. Valve 1 connects the vacuum chamber to the outside environment. Valve 2 connects the vacuum chamber to a vacuum pump, which is not shown in the figure. The MSA-400 laser Doppler vibrometer was used to measure the velocity of the vibrating channel through the glass window. The actuation voltage was applied to the device using a vacuum feedthrough for electronic connections.

Continuum regime measurements were performed between 0.1 bar and 1 bar. The pressure inside the chamber was measured with a Bronkhorst IQ+ FLOW pressure sensor instead of the pressure gauge shown in Figure 10. Valve 1 was used to control the pressure inside the chamber. The Q factor was again measured with the ring down method using the MSA-400 laser Doppler vibrometer to record the instantaneous velocity as a function of time. This measurement was only performed for the swing mode and twist mode of the device in Figure 1c.

Molecular flow regime measurements were performed between 0.015 mbar and 0.87 mbar. In this regime, the Q factors are much higher. Therefore, instead of using the ring down method, the MSA-400 laser Doppler vibrometer was used to apply a frequency sweep to the piezoelectric thin film actuators, and the Q factor was determined from the frequency response. This measurement was only performed for the device shown in Figure 1c operated in swing mode.

## 5. Results and Discussion

### 5.1. Q Factor at Atmospheric Pressure

**Figure 11:**
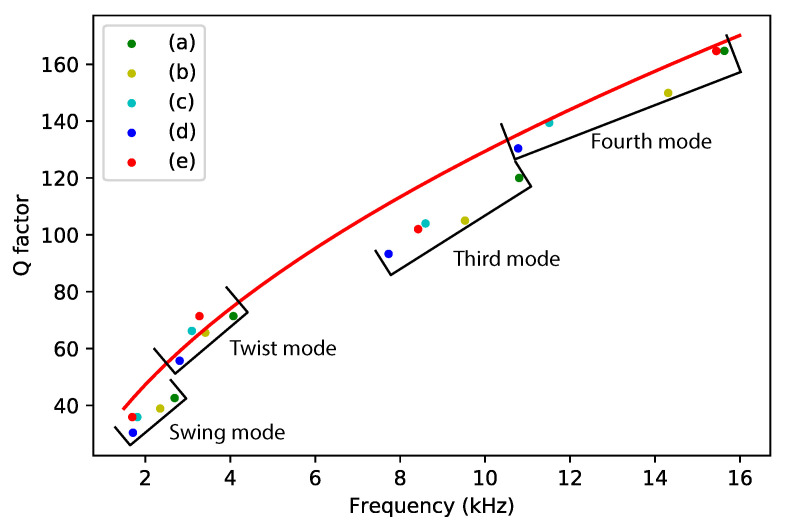
Measured and theoretically predicted Q factor as a function of resonance frequency. Measurement points with the same color represent measurement results from the same device. The red line represents the Q factor calculated from Equation (24).

Table 3 shows a summary of the measured Q factors for different devices at different vibration modes. Figure 11 shows a plot of the measured Q factors as a function of resonance frequency together with the prediction from Equation (24).

The prediction matches the measured quality factors very well. The prediction from (24) should overestimate the Q factor because the additional damping due to the capacitive readout electrodes is not included. However, we see that some measured quality factors are slightly higher than the prediction. This might be due to variations in the diameter of the channel. The measurement results for the swing mode and the third mode are significantly below the predicted values. These modes are affected the most by damping due to the capacitive readout electrodes. A more precise calculation based on Equation (22) is shown in Table 4.

The Q factors calculated from Equation (22) are very close to the measured Q factors in Table 3 with an error less than 15%. This indicates that the extra damping due to the capacitive readout combs indeed explains the lower quality factor in the swing and third mode. Table 4 also shows that the ratio $R_{kinetic}$ is at least an order of magnitude higher for the swing mode and third mode compared to the twist mode and fourth mode, which was already expected based on the mode shapes of the four modes.

### 5.2. Q Factor at Pressures from 0.1 Bar to 1 Bar

**Figure 12:**
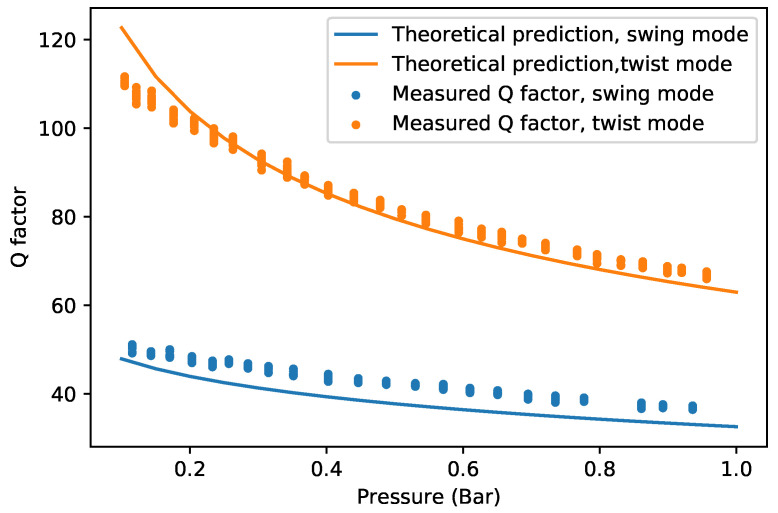
Measured and theoretically predicted Q factor of device (c) for the swing mode as a function of pressure.

Figure 12 shows the measured swing mode and twist mode Q factor of device (c) in the continuum regime of low vacuum. The theoretically predicted Q factor is slightly lower than the measured result. The trend matches well with the measurement results between 0.3 bar and 1 bar. However, the measured Q factors are smaller than predicted at lower pressures. The difference between the measured Q factor and the predicted Q factor might be due to the influence of the silicon substrate underneath the channel. When pressure decreases, the density of gas decreases while the viscosity remains unchanged. According to Equation (6), with decreasing pressure, δ increases, and the influence of the silicon chip body can no longer be ignored.

### 5.3. Q factor in the Molecular Flow Regime

**Figure 13:**
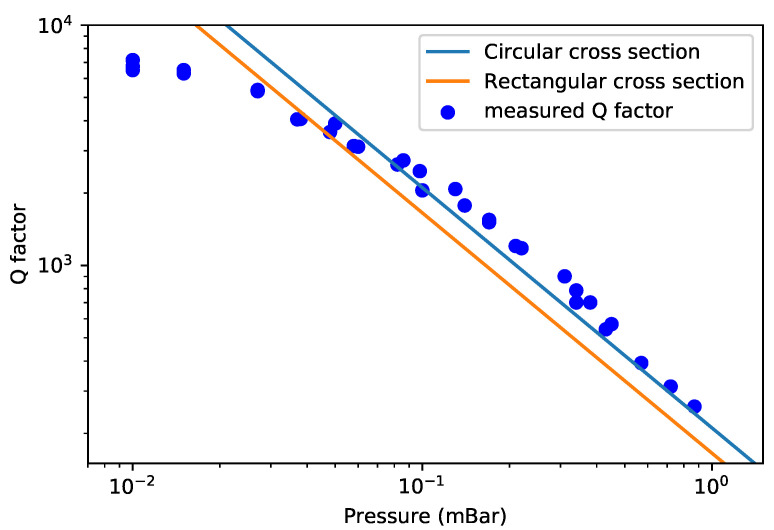
Measured and theoretically predicted Q factor for the swing mode in the molecular flow regime.

Figure 13 shows the measured Q factors together with the theoretically predicted Q factors based on Equation (25). The measurement was performed in a pressure range from 0.01 mbar to 0.87 mbar. The resulting Q factor ranges from 7155 to 258.

The measured Q factor is inversely proportional to the pressure and roughly follows the theoretical prediction, although the predicted value is slightly lower than the measurement result. The measured Q factors are closer to the theoretical prediction using circular cross sections. The most probable source of the remaining difference is the line density of the channel. The line density of the channel might be slightly higher than the value used in calculation. The measured Q factor is smaller than the predicted value for pressures below 0.03 mBar because intrinsic damping becomes the dominant damping factor. We can conclude that $Q_{intrinsic}$ in Equation (2) is above 7200. This indicates that the damping induced by intrinsic effects such as thermoelastic damping and the direct piezoelectric effect of the piezoelectric actuators induce a $Q_{intrinsic}$ that is larger than this value. The capacitive readout structures do not induce any significant damping in the molecular flow regime.

## 6. Conclusions

In this paper, we applied theoretical models for the air damping of resonant structures to a micro-Coriolis mass flow sensor. For the design of the micro-Coriolis mass flow sensors operating in air at atmospheric pressure, a good prediction of the quality factor is important as it influences the optimal choice for the resonance frequencies of the device, which determines the sensitivity to mass flow. In this paper, analytical models were derived to predict the drag force acting on the vibrating channel as a function of the resonance frequency, mode shapes, and pressure. Finite element simulations were used to obtain an estimate for the additional damping due to the presence of capacitive readout electrodes. The models were validated with measurements using 5 different devices operated in 4 different vibration modes, which allowed the measurement of the quality factor at 20 different resonance frequencies in the range from 1.8 kHz to 16 kHz.

The pressure dependence of the Q factor was measured both in the continuum regime and in the free molecular regime. In an atmospheric environment, the Q factor is strongly related to the resonance frequency of the measured mode, the channel line density, and the width of the channel. For the same device, the Q factor increases when the mode number increases. For different devices resonating in the same mode, the device with higher resonance frequency typically has a higher Q factor. Sader’s model [10] gives a rough estimation of the Q factor in different resonant modes. Extra damping induced by capacitive readout combs should be taken into account for a more accurate estimation of the Q factor in case that the vibration amplitude at the position of the electrodes is relatively large. The theoretical calculation based on a circular cross section matches with the measurement results without considering the influence of the silicon substrate underneath. Between 0.1 bar and 1 bar, the Q factor increases slower than the simplified expression [12] when the pressure decreases. In the molecular flow regime, the Q factor is inversely proportional to the pressure. The quantitative results presented in this paper help to predict the Q factor with resonance frequency. The results also show that the upper limit of the Q factor is around 7000. This high Q factor is reached at pressures below $1\times 10^{-2}$ mbar. The obtained results can also be of interest for cantilevers or other suspended micro channels that resonate in air.

## Figures and Tables

**Table 1:** Flow regime, corresponding Knudsen number $K_{n}$, and calculated pressure based on characteristic length $L_{C}$ of 70μm.

Regimes	$K_{n}$ Range	Pressure (mbar)
Free molecular regime	>10	<0.01
Transitional flow regime	0.1–10	0.01–9.8
Slip flow regime	0.01–0.1	9.8–98
Continuum regime	<0.01	>98

**Table 2:** Value of parameters involved in simulations and calculations.

Parameters	Value
Line density of channel $\rho_{l}$	$8.58\times 10^{-4}$ g $m^{-1}$
Channel width $L_{c}$	70 μm
Density of air ρ	1.2kg/$m^{3}$
Viscosity of air μ	$1.81\times 10^{-5}$kg/(m · s)
Molecular weight of air	28.97 g ${\mathrm{mol}}^{-1}$
Interdigitated electrode width $W_{e}$	40 μm
Distance between electrodes $d_{e}$	115 μm

**Table 3:** Measured resonance frequencies/measured Q factors for different devices and vibration modes.

Device Index	Swing Mode	Twist Mode	Third Mode	Fourth Mode
(a)	2691.2Hz/42.6	4074.1Hz/71.4	10,802 Hz/120	15,631.8 Hz/164.7
(b)	2352.3Hz/38.9	3414.1Hz/65.6	9526 Hz/105.0	14,309.7 Hz/149.9
(c)	1813.7Hz/35.9	3097.1Hz/66.2	8597.6Hz/104.7	11,510.4 Hz/139.4
(d)	1710.8Hz/30.4	2809.2Hz/55.7	7728.0Hz/93.3	10,777.6 Hz/130.4
(e)	1694.3Hz/35.9	3275.4Hz/71.4	8423Hz/102.0	15,442.4 Hz/164.65

**Table 4:** Calculated $R_{kinetic}$ (21) / calculated Q factor (22) for different devices and vibration modes.

Device Index	Swing Mode	Twist Mode	Third Mode	Fourth Mode
(a)	$1.26\times 10^{-1}$/43.6	$1.10\times 10^{-3}$/74.70	$4.95\times 10^{-2}$/126	$1.46\times 10^{-3}$/168
(b)	$1.15\times 10^{-1}$/40.2	$9.73\times 10^{-4}$/66.8	$5.15\times 10^{-2}$/116	$1.57\times 10^{-3}$/159
(c)	$1.17\times 10^{-1}$/32.9	$1.04\times 10^{-3}$/62.7	$3.66\times 10^{-2}$/111	$9.83\times 10^{-4}$/140
(d)	$1.10\times 10^{-1}$/31.9	$8.67\times 10^{-4}$ /58.9	$3.58\times 10^{-2}$/104	$1.09\times 10^{-3}$/135
(e)	$1.26\times 10^{-1}$/30.7	$8.53\times 10^{-4}$/65.1	$5.21\times 10^{-2}$/107	$1.91\times 10^{-3}$/166

## Data Availability

All raw data used in this paper is available on request.
